# Gut microbial metabolites in inflammation-associated colorectal cancer: mechanisms and therapeutic implications

**DOI:** 10.3389/fmicb.2026.1802686

**Published:** 2026-05-01

**Authors:** Yafang Li, Qinglong Wu, Jing Zhang, Qingqing Xun

**Affiliations:** 1 School of Clinical Medicine, Jining Medical University, Jining, China; 2 Department of Gastroenterology, Affiliated Hospital of Jining Medical University, Jining Medical University, Jining, China; 3 Clinical Medical Research Center, Affiliated Hospital of Jining Medical University, Jining Medical University, Jining, China

**Keywords:** colitis-associated colorectal cancer, gut microbiota metabolites, microbiota intervention, secondary bile acids, short-chain fatty acids, targeted therapy, tumor microenvironment

## Abstract

Colitis-associated colorectal cancer (CAC) represents the most severe malignant complication of inflammatory bowel disease (IBD), characterized by high invasiveness, frequent recurrence, and poor prognosis. Recent studies have revealed that gut microbial metabolites are involved in the initiation and progression of CAC through a “metabolite-signaling pathway-epigenetics” regulatory network, demonstrating a remarkable dual modulatory role. Protective metabolites, such as short-chain fatty acids (SCFAs), tryptophan derivatives (e.g., indole compounds), and vitamin B2, exert anti-inflammatory, antioxidant, intestinal barrier-enhancing, and oncogenic pathway-suppressing effects. In contrast, pathogenic metabolites promote carcinogenesis. Prominent among such metabolites are secondary bile acids [e.g., deoxycholic acid (DCA)], trimethylamine N-oxide (TMAO), and high concentrations of hydrogen sulfide (H_2_S). These metabolites activate nuclear factor κB (NF-κB), stimulate the release of pro-inflammatory cytokines, induce DNA damage, and disrupt immune homeostasis. Conversely, elevated plasma TMAO levels are closely associated with poor survival outcomes, with high-risk individuals showing significantly increased risks of all-cause mortality and recurrence. This review systematically summarizes the microbial origins, dual mechanisms in CAC, and potential therapeutic value of several key gut microbiota-derived metabolites, including SCFAs, succinate, secondary bile acids, TMAO, tryptophan metabolites, polyamines, H_2_S, and vitamin B2. Based on current evidence, intervention strategies are primarily focused on regulating microbial metabolic balance via probiotics/prebiotics, increasing precursor supply of beneficial metabolites through dietary fiber supplementation, reconstructing gut homeostasis via microbiota transplantation, and developing metabolite analogs or chelators for targeted intervention. Although gut microbial metabolites offer new paradigms for early diagnostic biomarkers and targeted therapies in CAC, clinical translation still faces several challenges, including interindividual microbial heterogeneity, establishment of causal relationships between metabolites and disease, and optimization of clinical intervention pathways, which require further research breakthroughs.

## 1 Introduction

Colorectal cancer (CRC) ranks as the third most incident malignant tumor globally and constitutes the second primary cause of cancer-associated mortality, exhibiting a persistent upward trend in disease burden (Sung et al., 2021). As one of the most severe complications of inflammatory bowel disease (IBD), colitis-associated colorectal cancer (CAC) accounts for approximately 10 to 15% of total mortality in IBD patients (Keller et al., 2019). Compared with sporadic CRC, CAC is typically characterized by higher aggressiveness, recurrence rates, and therapeutic resistance, resulting in a poorer prognosis for patients. Regarding pathological mechanisms, IBD-associated CRC does not arise abruptly; rather, chronic inflammation serves as the key driver, following the classic stepwise carcinogenesis model of “chronic inflammation → dysplasia → colorectal cancer.” In this process, the sustained activation of the nuclear factor κB (NF-κB) signaling pathway acts as the core driving force. For example, butyrate exerts anti-inflammatory effects by directly blocking NF-κB nuclear translocation and transcriptional activity through inhibiting IκB kinase (IKK) activation, reducing inhibitor of nuclear factor κB (IκBα) degradation, suppressing p65 Ser536 phosphorylation, and enhancing p65 acetylation via its function as a histone deacetylase (HDAC) inhibitor (Inan et al., 2000). Upon activation, NF-κB directly triggers the secretion of pro-inflammatory cytokines, including tumor necrosis factor-α (TNF-α), interleukin-1β (IL-1β), and interleukin-6 (IL-6). These mediators not only maintain the local chronic inflammatory milieu but also disrupt normal cellular physiology and proliferation control by suppressing apoptosis and upregulating cyclin expression, thereby culminating in tumorigenesis and cancer progression (Grivennikov et al., 2010). To prevent excessive immune responses from damaging its own tissues, the body synchronously activates a sophisticated negative feedback regulatory mechanism, with interleukin-10 (IL-10) being one of the most critical players (Iyer and Cheng, 2012). IL-10 is a cytokine with potent anti-inflammatory and immunosuppressive functions. It not only prevents overactivation of the immune system, thereby avoiding severe damage to host tissues caused by inflammatory responses, but also mitigates intestinal inflammation by inhibiting the secretion of pro-inflammatory cytokines, such as TNF-α and interferon-γ, thus reducing the risk of CAC (Kessler et al., 2017). The underlying mechanism involves the selective suppression of macrophage-derived IL-12, effectively arresting the expansion of Th1-mediated immunity. This action skews the immune equilibrium toward a T helper 2 (Th2)/regulatory T cell-dominant state, consequently mitigating self-tissue injury resulting from hyperactive immune responses (Mirsepasi-Lauridsen et al., 2019). Simultaneously, it creates favorable conditions for the development of humoral immune responses driven by Th2-type cytokines such as IL-4 and IL-5, ultimately achieving directed remodeling of the immune response type. Animal studies have further confirmed that IL-10-deficient mice develop distal colitis after colonization with non-pathogenic *Escherichia coli* (Kim et al., 2007). These mice exhibited not only elevated levels of the Th1 cytokine interferon-γ but also aberrant overexpression of the Th2 cytokine IL-4. These findings reveal that the Th1/Th2 imbalance caused by IL-10 deficiency is a critical link in the induction of colitis, highlighting the essential role of IL-10 in maintaining immune homeostasis. The anti-inflammatory effect of IL-10 is crucial for controlling tissue damage, and a significant common pathway in inflammatory injury is oxidative stress—a pathological process driven by excessive reactive oxygen species (ROS). ROS are a class of oxygen-derived molecules with high chemical reactivity. At appropriate concentrations, ROS can serve as a rapid defensive weapon of the innate immune system to eliminate invading pathogens. However, sustained high concentrations of ROS lead to severe oxidative DNA damage, such as 8-oxoguanine formation, as well as single-strand and double-strand breaks. If these lesions are not promptly repaired by the Base Excision Repair system, they can subsequently trigger oncogenic mutations in key tumor suppressor genes, including adenomatous polyposis coli and p53 (Renaudin, 2021). Moreover, ROS indirectly activate the IKK complex, thereby facilitating the nuclear translocation of NF-κB and triggering the transcription of diverse pro-inflammatory mediators. This cascade not only intensifies the inflammatory milieu but also fuels aberrant cellular proliferation, culminating in a self-perpetuating “inflammation-ROS-NF-κB” positive feedback circuit that drives tumorigenesis (Min et al., 2022).

Recent studies have shown that gut microbiota metabolites profoundly influence the progression of CAC through a “metabolite-signaling pathway-epigenetics” ternary regulatory network, exhibiting significant dual-directional effects (Table 1). The gut microbiome encodes over 12 million genes, and its metabolites—such as short-chain fatty acids (SCFAs), secondary bile acids, and tryptophan derivatives—can directly act on intestinal epithelial cells, immune cells, and cancer stem cells, impacting inflammatory intensity, DNA damage repair, and cell fate determination (Luo et al., 2025; Tierney et al., 2019). Certain metabolites exhibit clear anti-cancer effects. For example, SCFAs (e.g., butyrate) exert anti-inflammatory and anti-tumor effects by inhibiting HDAC, promoting IL-10 secretion, and facilitating the differentiation of regulatory T cells (Tregs) (Sun et al., 2024). In contrast, certain gut microbiota metabolites exhibit cancer-promoting potential. For instance, genotoxins produced by specific *Escherichia coli* strains under anaerobic conditions, such as colibactin, possess the ability to directly alkylate DNA. This action induces DNA double-strand breaks and mutations, thereby promoting malignant proliferation (Wilson et al., 2019). Furthermore, certain metabolites exhibit bidirectional regulatory properties. For instance, deoxycholic acid (DCA) promotes inflammation and tumor progression by activating the NF-κB signaling pathway at low-to-moderate concentrations, whereas it induces apoptosis at high concentrations. Tryptophan metabolites (e.g., indole-3-propionic acid) can alleviate chronic inflammation by activating the aryl hydrocarbon receptor (AhR); however, their metabolic intermediates may also generate carcinogenic substances that drive the progression of CRC. This complexity in metabolism-host interactions poses new challenges for the precise intervention of CAC.

**Table 1:** Main sources, mechanistic pathways, and corresponding biological functions of gut microbiota-derived metabolites.

Metabolite	Source	Mechanistic pathway	Supporting evidence	Biological function	References
SCFAs	*Bifidobacterium*; *Faecalibacterium prausnitzii*	Activate the Wnt pathway; Inhibit NLRP3; Inhibit HDAC	AOM/DSS	Exert anti-inflammatory effects	Guo et al. (2020), Hu M. et al. (2022), Ji et al. (2016), Koh et al. (2016), Swafford et al. (2020), Zheng et al. (2025)
		Binds to GPR109A and activates NRF2	AOM/DSS	Inhibit tumorigenesis	
		Activates STAT6/PPAR-γ signaling	DSS	Promotes intestinal epithelial repair	
Succinate	Bacteroidetes; Firmicutes	Activation of NLRP3 via SUCNR1; Activation of NF-κB via SUCNR1	IBD-associated; AOM/DSS	Promote inflammation	Bauset et al. (2022), Dai et al. (2025), Li C. et al. (2024), Selak et al. (2005)
		Stabilization of HIF-α by blocking its degradation promotes the expression of genes such as VEGF, Snail, and LDHA	AOM/DSS	Promote tumorigenesis	
Secondary bile acids	Firmicutes; Actinobacteria	DCA induces NF-κB activation via the receptors TGR5	DSS	Promote inflammation	Collins et al. (2023), Farhana et al. (2016), Feng et al. (2022), Kim et al. (2017), Pai et al. (2004), Zhang et al. (2021)
		DCA destabilizes the E-cadherin/β-catenin complex, thereby activating β-catenin-mediated transcription	*In vitro* cell experiment	Promote tumorigenesis	
		LCA inhibits the G1/S transition; Activation of the Wnt/β-catenin signaling pathway promotes the expression of c-Myc, Cyclin D1, and LGR5	*In vitro* cell experiment	Impair the intestinal/gut barrier	
		UDCA activates the cAMP/PKA pathway and inhibits YAP-driven transcription; UDCA attenuates oxidative stress by suppressing Nrf2 signaling	AOM/DSS	Inhibit tumorigenesis	
TMAO	*Anaerococcus*; *Clostridium*	Inhibition of the mitochondrial electron transport chain, induction of ROS, and activation of NF-κB	*In vitro* cell experiment	Promote inflammation	Fang et al. (2025), Jalandra et al. (2021), Yan et al. (2025)
		Activation of the AMPK and PI3K/Akt/mTOR signaling axis promotes the Warburg effect in CAC cells	Human CRC cohorts without inflammatory background	Promote tumorigenesis	
Tryptophan metabolites	*Escherichia coli*	Induces NF-κB signaling; Inhibition of ferroptosis through activation of the Aldehyde dehydrogenase 1 family member A3-FSP1-coenzyme Q10 axis	DSS; Apc^Min/+^	Exert anti-inflammatory effects	Cui et al. (2024), Li and Young (2013), Liu M. et al. (2023)
Polyamines	*Clostridium*; *Lactobacillus*	Chromatin remodeling-mediated upregulation of CD44 expression promotes immunosuppressive microenvironment shaping and augments tumor cell autonomous malignancy	Apc^Min/+^	Promote tumorigenesis	Zhao et al. (2021)
H_2_S	Sulfate-reducing bacteria	Inhibiting cytochrome c oxidase to induce ROS and trigger oxidative stress; Reduce the secretion of MUC2 mucin	*In vitro* cell experiment; AOM/DSS	Promote tumorigenesis	Blachier et al. (2019), Hanna et al. (2025), Munteanu et al. (2024)
Vitamin B2	*Lactobacillus*; *Bifidobacterium*	Riboflavin, via its coenzymatic forms FMN and FAD, inhibits CerS3 activity to reduce very-long-chain ceramide synthesis and promote epithelial repair, while simultaneously activating the EGFR-PI3K/Akt/mTOR signaling pathway to enhance intestinal epithelial proliferation	AOM/DSS	Promote intestinal epithelial repair	LeBlanc et al. (2013), Qu et al. (2025)

Although several reviews have summarized gut microbial metabolites in CRC in recent years, this review innovatively proposes a testable mechanistic hypothesis: the bidirectional regulation of core microbial metabolites is governed by the “metabolite-signaling transduction-epigenetics” axis. This hypothesis transcends traditional descriptive summaries and establishes a predictive framework that can be validated in preclinical models and clinical cohorts, providing a novel theoretical basis for precise interventions in CAC. The complexity of the aforementioned metabolite-host interactions poses new challenges for precise intervention in CAC. To systematically review the research progress in this field, this article aims to comprehensively elucidate the dual regulatory roles of gut microbial metabolites in CAC. Attention will be focused on dissecting the microbial origins and key metabolic signaling pathways mediated by core metabolites, including SCFAs, secondary bile acids, and trimethylamine N-oxide (TMAO). Moreover, this review will provide a detailed mechanistic insight into how these metabolites drive tumorigenesis and cancer progression. Specifically, we will examine their capacity to modulate epigenetic landscapes—via DNA methylation and histone acetylation—and to orchestrate pivotal oncogenic signaling cascades, such as NF-κB, signal transducer and activator of transcription 3 (STAT3), and Wnt/β-catenin. Furthermore, building upon these insights, we will discuss emerging therapeutic paradigms leveraging the “metabolism-immune crosstalk.” Our goal is to offer a robust theoretical framework and actionable translational avenues to advance early detection, molecular stratification, and precision therapeutics for CAC.

## 2 Types of gut microbiota metabolites and their action mechanisms

### 2.1 SCFAs

SCFAs are core metabolic mediators produced by the gut microbiota through the fermentation of dietary fiber (Xiong et al., 2022). They primarily include acetate, propionate, and butyrate, each containing fewer than six carbon atoms. The production of these metabolites exhibits distinct genus-specificity: acetate is mainly produced by bacteria such as *Bifidobacterium*; propionate is primarily generated by bacteria like *Prevotella*; while butyrate is predominantly synthesized by Firmicutes bacteria, including *Faecalibacterium prausnitzii* (Koh et al., 2016). Among them, butyrate serves as the primary energy source for colonic epithelial cells, supplying 60–70% of their required energy, and demonstrates the highest bioactivity in maintaining gut homeostasis. SCFAs are not only energy substrates for the host but also serve as critical signaling molecules facilitating cross-kingdom communication between the gut microbiota and the host.

Recent studies indicate that SCFAs profoundly influence host health through multi-level mechanisms, with their core functions categorizable into three dimensions. First is anti-inflammatory action and barrier maintenance: SCFAs effectively alleviate intestinal inflammatory responses and inhibit epithelial cell apoptosis, thereby preserving the structural integrity of the gut barrier. Second is immune regulation and tolerance induction: they promote the differentiation of induced Tregs, enhance the body’s immunosuppressive functions, and prevent excessive immune activation. Finally, there is the antioxidant and anti-cancer effect: SCFAs scavenge excess ROS, protect DNA from oxidative damage, and consequently significantly reduce the risk of CRC.

**Figure 1:**
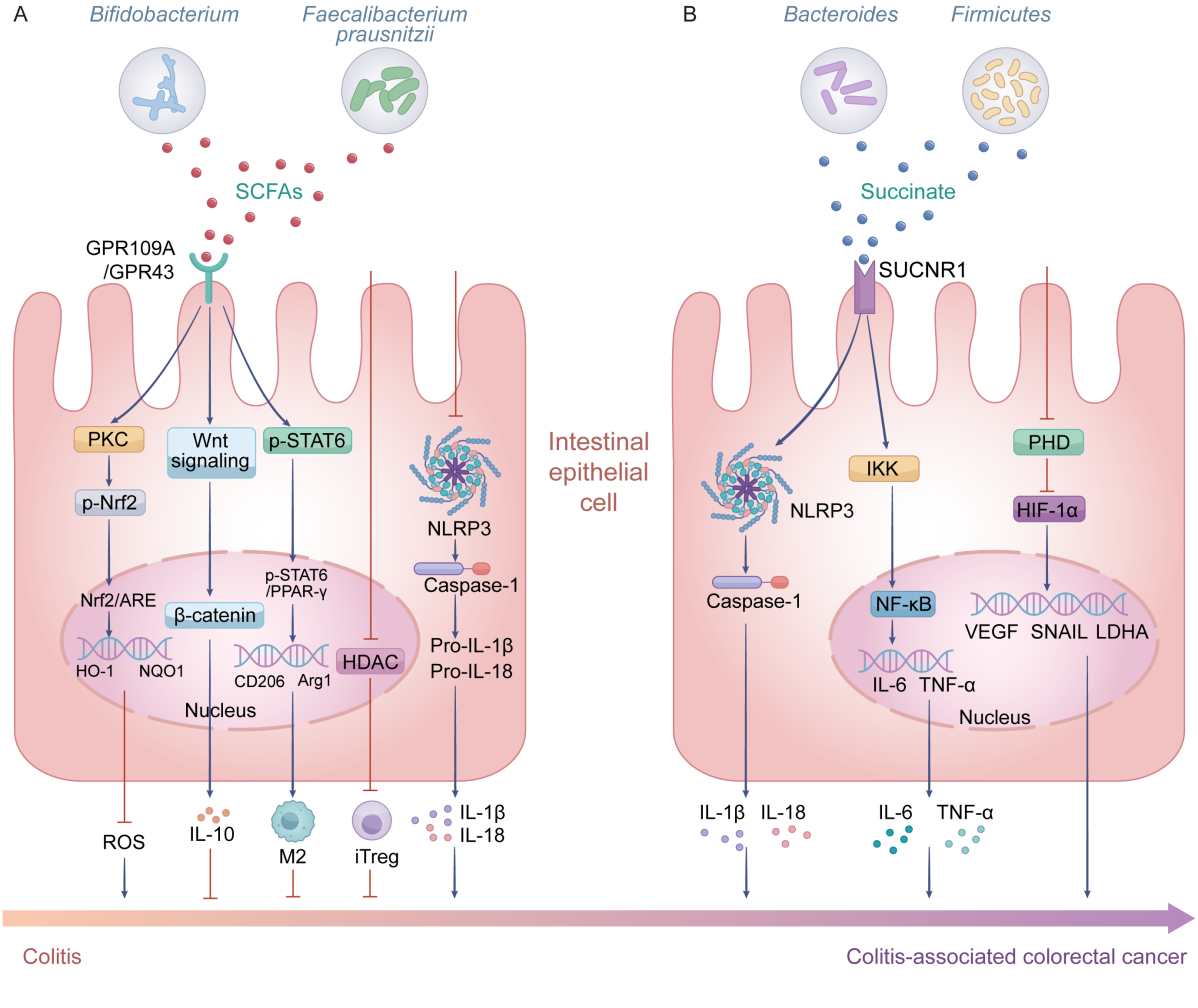
Core mechanisms by which short-chain fatty acids (SCFAs) and succinate mediate opposing effects in Colitis-associated colorectal cancer (CAC). **(A)** The inhibitory role of SCFAs, which are primarily produced by *Bifidobacterium* and *Faecalibacterium prausnitzii*. SCFAs act on intestinal epithelial cells via G-protein-coupled receptor 109A (GPR109A) and G-protein-coupled receptor 43 (GPR43) to activate protein kinase C (PKC)/nuclear factor erythroid 2-related factor 2 (Nrf2)-mediated antioxidant responses, including heme oxygenase-1 (HO-1) and NAD(P)H:quinone oxidoreductase 1 (NQO1), thereby reducing reactive oxygen species (ROS); concurrently, they promote the production of anti-inflammatory interleukin-10 (IL-10) through Wnt/β-catenin signaling pathway and facilitate the polarization of anti-inflammatory M2 macrophages via the signal transducer and activator of transcription 6 (STAT6)/peroxisome proliferator-activated receptor γ (PPAR-γ) pathway. In addition, independently of these receptors, SCFAs promote the generation of inducible regulatory T cells (Tregs) by inhibiting histone deacetylase (HDAC) signaling, and suppress NOD-like receptor family pyrin domain containing 3 (NLRP3) inflammasome activation as well as the secretion of pro-inflammatory interleukin-1β (IL-1β) and interleukin-18 (IL-18). Collectively, these actions alleviate colitis and inhibit the development of CAC. **(B)** In contrast, succinate derived from *Bacteroides* and Firmicutes activates succinate receptor 1 (SUCNR1), which triggers the NLRP3 inflammasome and IκB kinase (IKK) pathways, leading to the production of pro-inflammatory cytokines such as IL-1β, IL-18, interleukin-6 (IL-6), and tumor necrosis factor-α (TNF-α). Furthermore, succinate promotes tumor progression during CAC development by upregulating the expression of vascular endothelial growth factor (VEGF), zinc finger transcription factor SNAIL (SNAIL), and lactate dehydrogenase A (LDHA), thereby facilitating angiogenesis, epithelial–mesenchymal transition, and metabolic reprogramming. Created with Adobe Illustrator.

The specific effects of SCFAs depend on the upstream signals they trigger and the subsequent downstream cascades. In terms of epigenetic regulation and immune tolerance, butyrate serves as a representative example: acting as a HDAC inhibitor, it suppresses HDAC activity to increase histone acetylation levels. This process stabilizes the expression of the key transcription factor Foxp3, thereby promoting the differentiation of Tregs and enhancing their immunosuppressive functions (Hu M. et al., 2022) (Figure 1A).

Meanwhile, butyrate also prevents the normal degradation of ubiquitin-tagged inhibitor of nuclear factor κB (IκBα) protein. This leads to the sustained inhibition of NF-κB activation, thereby blocking the transcription and translation of inflammatory genes at their source. In terms of receptor-mediated anti-inflammatory and antioxidant pathways, SCFAs act through G-protein-coupled receptor 109A (GPR109A) and G-protein-coupled receptor 43 (GPR43) to inhibit the assembly and activation of the NOD-like receptor family pyrin domain containing 3 (NLRP3) inflammasome. This reduces the maturation and release of IL-1β and IL-18, thereby alleviating local inflammation (Figure 1A). Furthermore, butyrate specifically binds to and activates GPR109A/GPR43 receptors, triggering protein kinase C (PKC) signaling that leads to the activation of the antioxidant transcription factor nuclear factor erythroid 2-related factor 2 (Nrf2) (Guo et al., 2020) (Figure 1A). This initiates the expression of antioxidant genes, facilitating the scavenging of ROS. In terms of barrier repair, SCFAs can also activate the Wnt signaling pathway, stabilizing β-catenin protein and promoting its nuclear translocation. This subsequently enhances the expression and secretion of IL-10, synergistically alleviating inflammation and inhibiting epithelial cell apoptosis (Swafford et al., 2020) (Figure 1A). Meanwhile, butyrate enhances the signal transducer and activator of transcription 6 (STAT6)/peroxisome proliferator-activated receptor γ (PPAR-γ) signaling pathway to drive the M2 genetic expression program and induce metabolic reprogramming, thereby promoting the polarization of M2 macrophages (Ji et al., 2016) (Figure 1A). This process not only helps maintain intestinal immune tolerance but also accelerates the repair of epithelial tissue following injury. It is worth noting that although endogenous acetate is typically regarded as a protective factor, *Alcaligenes* species constitute a significant counterexample. These bacteria can abnormally secrete excessive amounts of acetate, which in turn activates key acetyltransferases (Zheng et al., 2025). This disrupts intercellular junctions, leading to barrier collapse and triggering sustained, severe inflammatory responses that ultimately drive the progression from colitis to CRC. These studies reveal the multifaceted regulatory roles of SCFAs within the gut microenvironment: their specific effects ultimately depend on the comprehensive integration of metabolite type, concentration, and local microenvironmental signaling networks.

### 2.2 Succinate

In the gut microbiota, succinate is predominantly synthesized by members of the phylum Bacteroidetes and the phylum Firmicutes (e.g., specific *Clostridium* species) via carbohydrate fermentation pathways (Dai et al., 2025). Recent studies indicate that gut microbiota dysbiosis (e.g., the increase in *Bacteroides* and decrease in *Clostridium* commonly observed in patients with IBD) leads to elevated succinate production, resulting in its accumulation in the intestinal lumen and systemic circulation (Anthamatten et al., 2024). During the development of CAC, succinate promotes inflammation and tumor progression through multiple mechanisms. Clinical studies have shown that fecal succinate levels are significantly elevated in patients with IBD and are positively correlated with CRC tumor stage and lymph node metastasis.

At the molecular mechanism level, succinate promotes intestinal inflammation and tumor progression through the following two major signaling axes. Succinate anchors key arginine and tyrosine residues on the receptor using its carboxyl groups at both ends, thereby driving the conformational rearrangement of hydrophobic residues within the receptor. This causes the outward movement of transmembrane helix 6, opening the intracellular G protein-binding pocket and thereby activating succinate receptor 1 (SUCNR1) (Li C. et al., 2024). On the one hand, by triggering the Gq/PLC signaling pathway and mitochondrial reverse electron transport-mediated ROS bursts, it induces the oligomerization and activation of the NLRP3 inflammasome, driving the maturation and secretion of pro-inflammatory cytokines such as IL-1β and IL-18, thereby significantly amplifying local intestinal inflammatory responses (Bauset et al., 2022) (Figure 1B). On the other hand, the ligand-receptor complex further activates the downstream NF-κB signaling pathway, significantly promoting the transcription, expression, and biosynthesis of key pro-inflammatory mediators, including IL-1β and TNF-α, thereby driving the initiation and cascade amplification of intestinal inflammation (Figure 1B). Succinate can also competitively inhibit the activity of prolyl hydroxylase (PHD), thereby preventing the normal prolyl hydroxylation and subsequent proteasomal degradation of hypoxia-inducible factor-1α (HIF-1α) protein under normoxic conditions (Selak et al., 2005). This leads to the abnormal stabilization and accumulation of HIF-1α in the cytoplasm. Subsequently, HIF-1α translocates into the nucleus, where it forms a transcriptional complex with HIF-β, activating a series of downstream target genes closely associated with tumor progression (Selak et al., 2005) (Figure 1B). This process systematically promotes malignant biological processes such as tumor angiogenesis, cell proliferation, metabolic reprogramming, and metastasis. In summary, gut microbiota-derived succinate primarily amplifies inflammation and drives the expression of pro-tumorigenic genes through the SUCNR1 and HIF-1α signaling axis, thereby promoting the occurrence and development of CRC in the context of IBD.

### 2.3 Secondary bile acids

**Figure 2:**
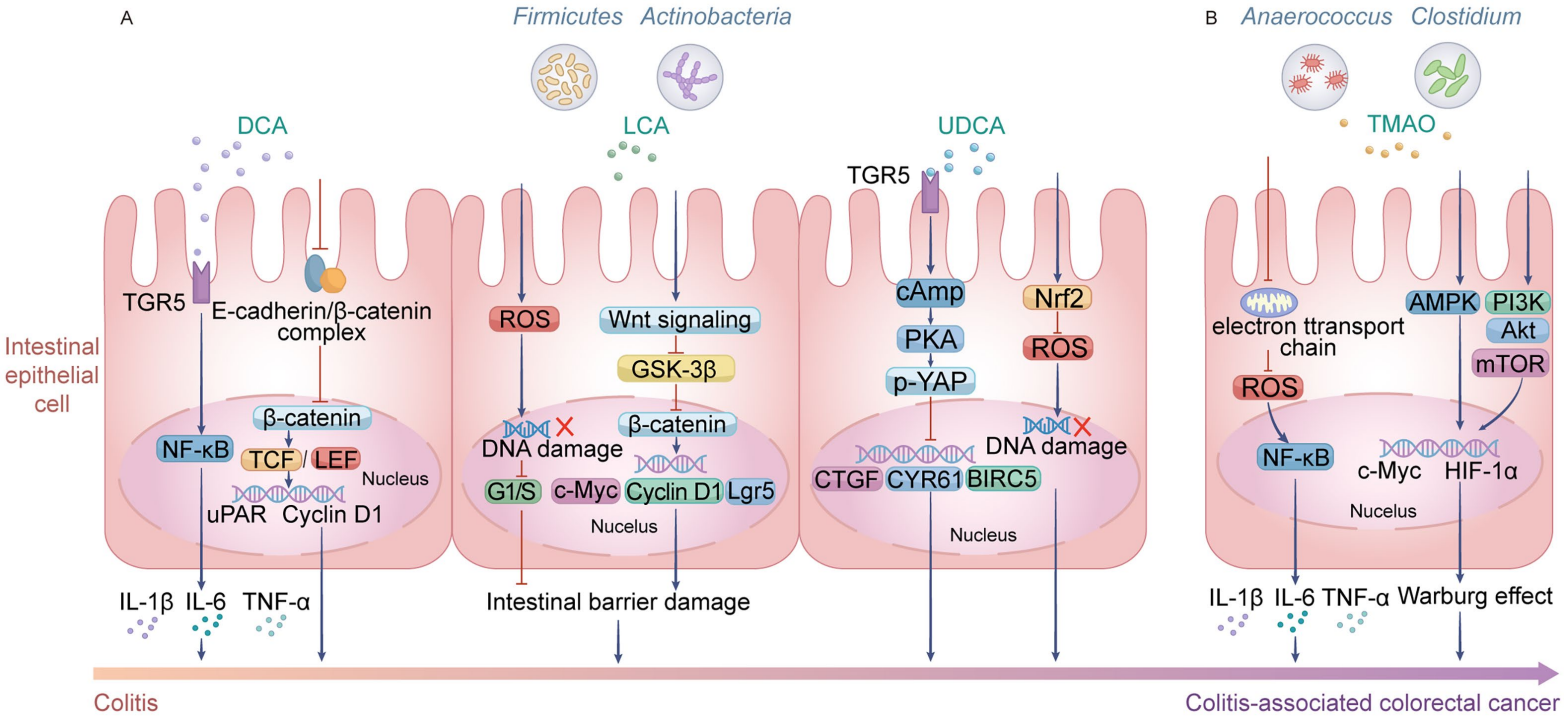
Core mechanisms of secondary bile acids and trimethylamine N-oxide (TMAO) driving CAC progression. **(A)** Secondary bile acids, produced by gut microbiota including *Firmicutes* and *Actinobacteria*, regulate CAC progression through distinct pathways: deoxycholic acid (DCA) activates the Takeda G protein-coupled receptor 5 (TGR5) and disrupts the E-cadherin/β-catenin complex, inducing nuclear translocation of nuclear factor κB (NF-κB) and β-catenin/T cell factor (TCF)/lymphoid enhancer-binding factor (LEF). This upregulates pro-inflammatory cytokines (IL-1β, IL-6, TNF-α) and oncogenes [Urokinase-type plasminogen activator receptor (uPAR), Cyclin D1], exacerbating colitis and promoting tumorigenesis. Lithocholic acid (LCA) induces accumulation of ROS, causing DNA damage and activating Wnt signaling via inhibition of glycogen synthase kinase-3β (GSK-3β). Subsequent nuclear β-catenin activity drives expression of c-Myc, Cyclin D1, and Lgr5, disrupting intestinal barrier integrity and accelerating CAC progression. UDCA exerts a modulatory role: TGR5-mediated cAMP/PKA signaling induces Yes-associated protein (YAP) phosphorylation, suppressing downstream target genes [connective tissue growth factor (CTGF), cysteine-rich angiogenic inducer 61 (CYR61), and baculoviral IAP repeat containing 5 (BIRC5)] to affect cell survival and proliferation; meanwhile, it antagonizes DNA damage through Nrf2 and ROS signaling, thereby inhibiting CAC development. **(B)** TMAO, metabolized by *Anaerococcus and Clostridium, pr*omotes CAC through multiple oncogenic pathways: TMAO inhibits mitochondrial electron transport chain activity and promotes ROS production, activating NF-κB to drive expression of pro-inflammatory cytokines (IL-1β, IL-6, TNF-α) and sustain chronic inflammation. It also activates AMP-activated protein kinase (AMPK) and phosphoinositide 3-kinase (PI3K)/Ak strain transforming (Akt)/mammalian target of rapamycin (mTOR) signaling, upregulates c-Myc and hypoxia-inducible factor-1α (HIF-1α) to induce the Warburg effect, enabling metabolic reprogramming and facilitating rapid tumor cell proliferation in CAC. Created with Adobe Illustrator.

The biosynthesis of secondary bile acids relies on a coordinated “liver synthesis–gut microbiota conversion” mechanism: the liver first utilizes cholesterol 7α-hydroxylase to catalyze the conversion of cholesterol into primary bile acids (e.g., cholic acid and chenodeoxycholic acid); subsequently, in the intestine, bacteria belonging to the phyla Firmicutes and Actinobacteria employ 7α-dehydroxylase enzymes to transform these primary bile acids into secondary bile acids, including DCA, lithocholic acid (LCA), and ursodeoxycholic acid (UDCA) (Collins et al., 2023). These three metabolites exhibit distinct bidirectional regulatory effects during the inflammatory carcinogenesis of CRC. Among them, DCA and LCA primarily function as pro-carcinogenic drivers. Their macroscopic phenotypic manifestations include inducing chronic inflammation, driving epithelial cell carcinogenesis, promoting tumor proliferation and invasion, inhibiting apoptosis, and compromising intestinal barrier integrity, ultimately leading to the abnormal survival and accumulation of potentially carcinogenic cells. In-depth mechanistic studies reveal that DCA primarily activates the Takeda G protein-coupled receptor 5 (TGR5) on the surface of intestinal epithelial and immune cells, triggering the NF-κB signaling pathway and inducing the release of pro-inflammatory cytokines such as IL-1β, IL-6, and TNF-α, while simultaneously inducing ROS bursts that lead to DNA damage (Figure 2A).

Furthermore, DCA disrupts the E-cadherin/β-catenin complex, enhances β-catenin transcriptional activity, and upregulates target genes including Urokinase-type plasminogen activator receptor and cyclin D1, thereby driving malignant progression (Figure 2A) (Pai et al., 2004). In contrast, LCA primarily inhibits apoptosis in damaged cells by disrupting mitochondrial function and mimics growth factors to activate the Wnt/β-catenin pathway, thereby maintaining stem cell characteristics and driving aberrant proliferation (Farhana et al., 2016) (Figure 2A). Studies have also found that LCA can arrest the cell cycle at the G1/S phase, further compromising intestinal barrier function and exacerbating the inflammatory microenvironment (Feng et al., 2022) (Figure 2A). In contrast, UDCA exhibits significant chemopreventive and anti-tumor properties, effectively inhibiting cancer cell proliferation and invasion while maintaining intestinal homeostasis. Its molecular mechanism involves a dual protective axis: on the one hand, UDCA activates the TGR5 receptor to mediate the cAMP/PKA signaling cascade, prompting the phosphorylation and cytoplasmic retention of the oncogenic transcriptional co-activator Yes-associated protein (YAP), thereby blocking YAP-driven oncogenic transcriptional programs (Zhang et al., 2021) (Figure 2A). On the other hand, UDCA specifically activates the nuclear factor Nrf2, promoting its nuclear translocation and binding to Antioxidant Response Elements (ARE). This initiates the expression of key antioxidant enzymes such as NAD(P)H:quinone oxidoreductase 1, heme oxygenase-1, and glutathione synthetase, comprehensively enhancing cellular defense capabilities against oxidative stress (Kim et al., 2017) (Figure 2A). In summary, secondary bile acids play a complex double-edged sword role in the initiation and progression of CRC by regulating key pathways including inflammatory signaling, DNA damage repair, cell cycle progression, and antioxidant defense.

### 2.4 TMAO

TMAO, a key end-product generated by gut microbiota (Anaerococcus, Clostridium, etc.) through the metabolism of dietary nutrients like choline and lecithin, and subsequently formed via catalysis by hepatic flavin-containing monooxygenases, has emerged as a crucial molecular link connecting gut dysbiosis to the initiation and progression of CRC (Jalandra et al., 2021). Existing research confirms that TMAO does not act through a single pathway, but rather drives tumor progression via synergistic multi-dimensional molecular mechanisms. First, regarding the construction of the inflammatory microenvironment, TMAO induces excessive production of ROS by inhibiting the mitochondrial electron transport chain and upregulating the expression of nicotinamide adenine dinucleotide phosphate oxidases (e.g., NADPH oxidase 4). This subsequently leads to the sustained activation of the NF-κB signaling pathway and promotes the transcription and translation of pro-inflammatory cytokines, including IL-1β, IL-6, and TNF-α (Figure 2B). Meanwhile, TMAO can specifically bind to the Caspase Recruitment Domain domain of the NLRP3 inflammasome to enhance its oligomerization. This amplification effect of oxidative stress and inflammatory cascades is clinically manifested as a significant positive correlation between serum TMAO levels and inflammatory factors such as IL-17A and IL-8 in patients (Fang et al., 2025). Secondly, TMAO remodels the tumor stroma by inducing cellular senescence. The underlying mechanism involves the upregulation of p16INK4a and p21Cip1 expression, which triggers cell cycle arrest via the p53/p21 and RB/E2F pathways and induces the senescence-associated secretory phenotype (Ke et al., 2018). The abnormally secreted factors, including IL-6, matrix metalloproteinase-3, and granulocyte-macrophage colony-stimulating factor, further activate cancer-associated fibroblasts through STAT3 phosphorylation, thereby promoting collagen deposition and angiogenesis. This process has been validated in both animal models and *in vitro* experiments (Li et al., 2022). Furthermore, TMAO severely compromises intestinal barrier integrity. It not only causes the displacement and disruption of tight junction proteins zonula occludens-1 and Claudin but also synergistically enhances bacterial High Temperature Requirement A protease-mediated cleavage of Occludin (Smith et al., 2024). Additionally, through multiple pathways—including ROS/NLRP3/Gasdermin D-mediated pyroptosis, endoplasmic reticulum stress, and autophagy inhibition—TMAO induces programmed death of intestinal epithelial cells (Liu et al., 2025). *In vivo* studies demonstrate that this barrier impairment directly leads to elevated serum lipopolysaccharide levels, establishing a cycle of “barrier disruption–bacterial translocation–chronic inflammation”. Furthermore, TMAO is deeply involved in epigenetic regulation and metabolic reprogramming. On the one hand, it activates the Wnt/β-catenin pathway by inhibiting DNA methyltransferase 1 activity, thereby inducing demethylation of the Secreted Frizzled-Related Protein 1 promoter. On the other hand, it competitively inhibits α-ketoglutarate-dependent dioxygenases, disrupting the balance of histone modifications. Additionally, TMAO promotes the Warburg effect via the MAPK and phosphoinositide 3-kinase (PI3K)/Akt/mammalian target of rapamycin (mTOR) axes, providing both the energy supply and epigenetic foundation for the malignant proliferation of cancer cells (Yan et al., 2025) (Figure 2B). In summary, through the synergistic effects of multiple pathways, including inflammatory activation, microenvironment remodeling, barrier function impairment, and metabolic-epigenetic reprogramming, TMAO establishes its central role in the pathogenic network of CRC.

### 2.5 Tryptophan metabolites

**Figure 3:**
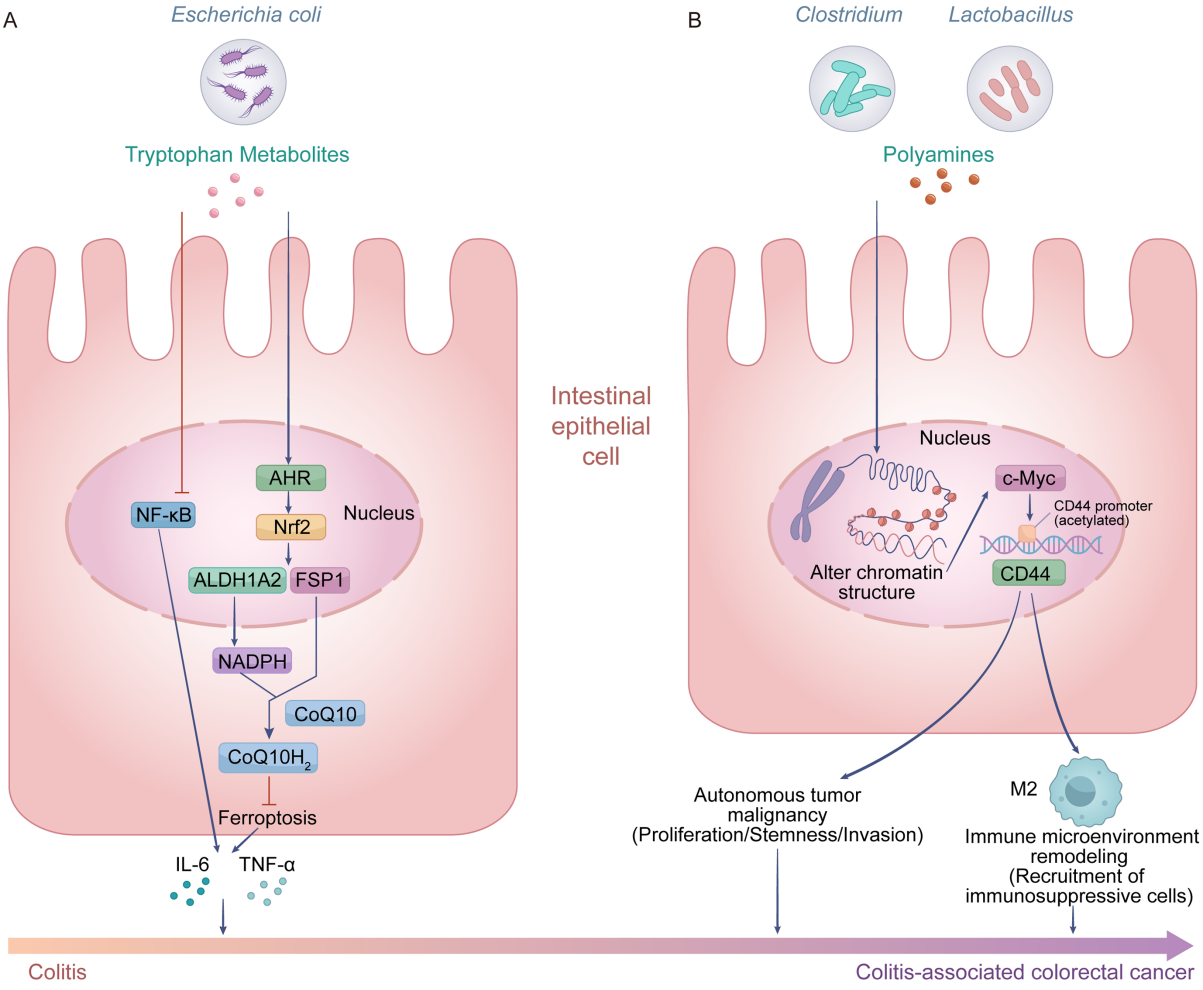
Dual roles of tryptophan metabolites and polyamines in the progression of CAC. **(A)** Tryptophan metabolites, derived from *Escherichia coli*, exert protective effects: they activate the aryl hydrocarbon receptor (AhR)/nuclear factor erythroid 2-related factor 2 (Nrf2) signaling axis, upregulate aldehyde dehydrogenase 1 family member A2 (ALDH1A2) and ferroptosis suppressor protein 1 (FSP1) expression, enhance nicotinamide adenine dinucleotide phosphate (NADPH)-dependent reduction of coenzyme Q10 (CoQ10) to its reduced form (CoQ10H_2_), and inhibit ferroptosis in intestinal epithelial cells; meanwhile, they suppress the NF-κB pathway, reduce production of pro-inflammatory cytokines IL-6 and TNF-α, alleviate colitis, and block the progression from chronic inflammation to CAC. **(B)** Polyamines, produced by *Clostridium and Lactobacillus, pr*omote CAC progression: they induce chromatin remodeling in intestinal epithelial cells, trigger acetylation of the Cluster of Differentiation 44 (CD44) promoter, and upregulate c-Myc-mediated CD44 expression. This process enhances tumor-autonomous malignant phenotypes (including proliferation, stemness, and invasiveness) and remodels the immune microenvironment by recruiting immunosuppressive M2 macrophages, collectively driving the initiation and progression of CAC. Created with Adobe Illustrator.

Tryptophan metabolites exhibit a complex double-edged sword characteristic in the occurrence and development of inflammatory bowel cancer, with their biological effects highly dependent on the balance status of the gut microbiota. Under steady-state conditions, gut *Escherichia coli* efficiently converts tryptophan into indole via tryptophanase. This conversion process is significantly enhanced in nutrient-rich environments with high concentrations of exogenous tryptophan (Li and Young, 2013). Mechanistic studies indicate that the gut microbiota metabolizes indole into indole-3-aldehyde. Indole-3-aldehyde effectively alleviates chronic inflammation of the colonic mucosa by inhibiting the activation of the NF-κB signaling pathway, thereby blocking the release of downstream pro-inflammatory cytokines, IL-6 and TNF-α (Liu M. et al., 2023) (Figure 3A).

Furthermore, animal experiments have confirmed that indole supplementation significantly reduces the incidence of inflammatory bowel cancer, establishing its pivotal role in maintaining intestinal barrier function and exerting anti-inflammatory and anti-tumor effects. However, when gut dysbiosis occurs, specific pathogenic bacteria can utilize the same substrate to generate indole derivatives with pro-carcinogenic activity, leading to a fundamental reversal in metabolic trajectory. For instance, *Fusobacterium nucleatum* metabolizes tryptophan to produce 3-indolepropionic acid, which enters macrophages and activates the AhR, inducing their polarization toward the M2 (pro-carcinogenic/anti-inflammatory) phenotype (Song et al., 2025). This subsequently aids CRC cells in evading host immune surveillance and accelerates tumor growth and progression. More critically, *Peptostreptococcus anaerobius*, as a dominant functional bacterium in the gut of patients with inflammatory bowel cancer, highly expresses key enzymes involved in tryptophan metabolism and produces large amounts of trans-3-indoleacrylic acid. As an endogenous ligand for the AhR, trans-3-indoleacrylic acid activates the downstream Aldehyde dehydrogenase 1 family member A3-ferroptosis suppressor protein 1 (FSP1)-coenzyme Q10 signaling axis (Cui et al., 2024) (Figure 3A). This mechanism not only inhibits cellular ferroptosis but also promotes the secretion of inflammatory cytokines such as IL-6 and TNF-α, thereby exacerbating the local inflammatory microenvironment. Clinical cohort studies strongly support this mechanism, revealing that both the abundance of this bacterium and the levels of its metabolites are significantly higher in the guts of patients with inflammatory bowel cancer compared to healthy individuals, accompanied by markedly enhanced activation of the AhR pathway in tumor tissues. Notably, patients harboring high abundances of this bacterium but exhibiting low serum metabolite concentrations demonstrated significantly prolonged survival, confirming the critical driving role of this metabolite in disease progression. To address the limitations of natural metabolites, FKK6, a microbial metabolic analog based on indole structural modification, demonstrates significant therapeutic potential (Sládeková et al., 2025). It specifically activates the AhR–IL-22 signaling axis, inducing intestinal epithelial cells to secrete the antimicrobial peptide Regenerating islet-derived protein 3γ. This action reshapes the composition of the gut commensal microbiota, successfully reversing the occurrence of colitis-associated tumors in animal models while circumventing the instability associated with natural products. In summary, differences in the composition of the gut microbiome are the fundamental determinant driving tryptophan metabolism toward divergent pathological outcomes. A high abundance of *Escherichia coli* tends to generate protective indoles, whereas the overproliferation of *Fusobacterium nucleatum* or *Peptostreptococcus anaerobius* leads to the accumulation of pro-carcinogenic derivatives. This inter-individual metabolic heterogeneity is further finely regulated by multiple factors, including host genetic polymorphisms, dietary habits, immune status, and disease stage. In-depth elucidation of the microbiota-mediated indole metabolic network and its individual variations reveals the dynamic switching mechanism between tumor suppression under microbial homeostasis and tumor promotion during dysbiosis. These insights provide a crucial theoretical foundation and novel targets for the precise prevention, risk stratification, and development of personalized intervention strategies based on prebiotics or small-molecule drugs for inflammatory bowel cancer.

### 2.6 Polyamines

Polyamines in the gut are primarily synthesized by bacterial genera such as *Clostridium* and *Lactobacillus* through amino acid precursors like ornithine and arginine (Zhao et al., 2021). In the progression of CAC, elevated fecal polyamine levels not only reflect the state of inflammatory hyperplasia during the IBD phase but also serve as critical metabolic molecules driving the inflammation-cancer cascade (Li et al., 2025; Yang et al., 2019). They achieve this by inducing genomic instability, sustaining a pro-tumorigenic inflammatory microenvironment, and mediating tumor immune evasion. Consequently, the polyamine metabolic pathway, acting as a pivotal molecular hub linking chronic intestinal inflammation to colorectal carcinogenesis, represents a potential target for the prevention and intervention of CAC. At the molecular mechanism level, polyamines first upregulate Cluster of Differentiation 44 (CD44) expression by remodeling chromatin structure, thereby enhancing the stemness and invasive/metastatic capacity of tumor cells. Simultaneously, polyamines reshape the immunosuppressive microenvironment, specifically manifested by the recruitment and activation of myeloid-derived suppressor cells and the induction of M2-type tumor-associated macrophage polarization, thus enabling tumors to evade immune surveillance (Figure 3B). The synergistic interplay of the aforementioned epigenetic regulation, immunosuppression, and pro-proliferative signaling collectively drives malignant tumor progression, positioning gut microbiota-derived polyamines as potential therapeutic targets.

### 2.7 Hydrogen sulfide

**Figure 4:**
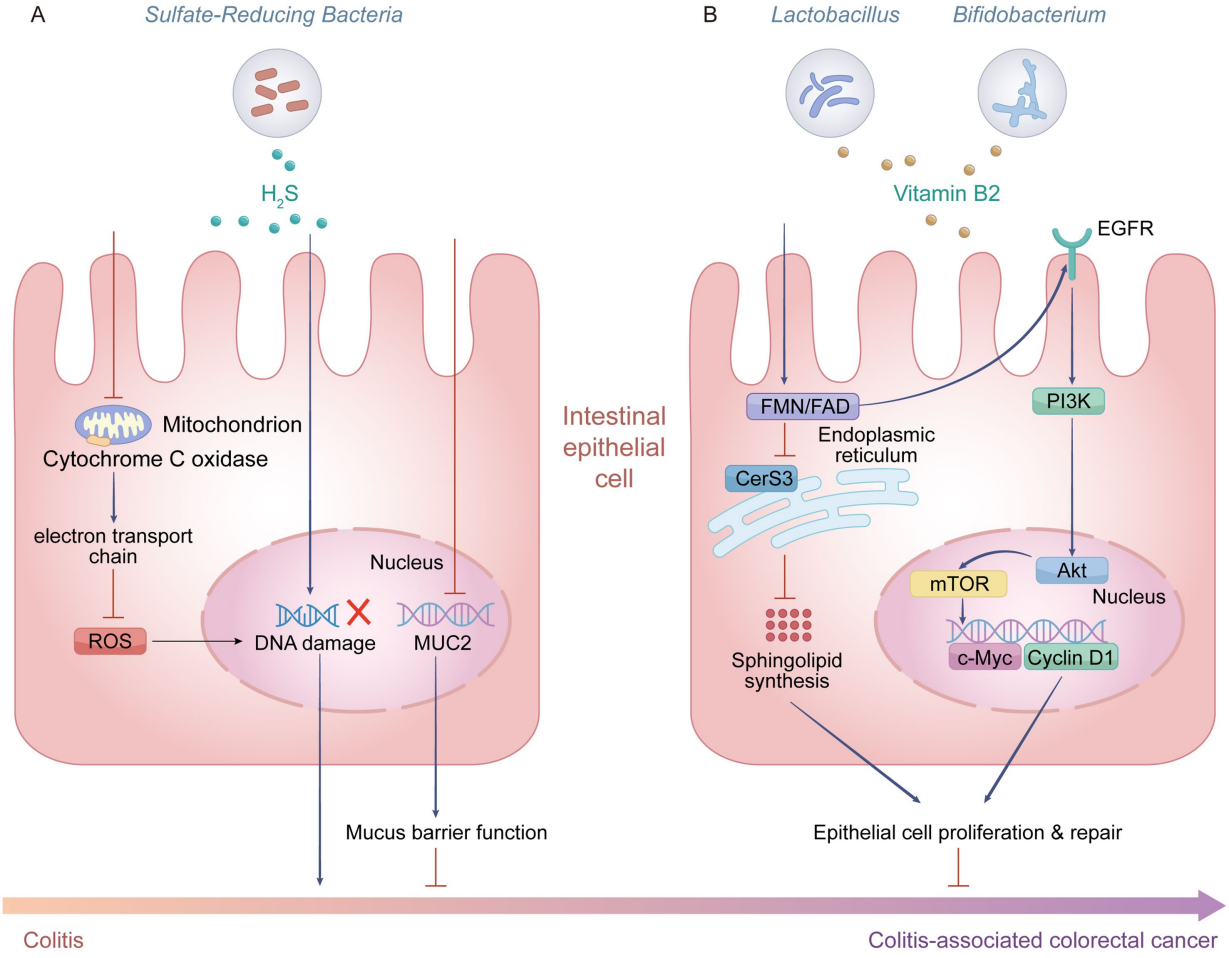
Schematic diagram of the mechanisms by which H_2_S and vitamin B2 regulate CAC progression. **(A)** High concentrations of hydrogen sulfide (H_2_S) produced by sulfate-reducing bacteria (SRB) exert pro-tumorigenic effects in intestinal epithelial cells: H_2_S inhibits mitochondrial cytochrome *c* oxidase, disrupts the electron transport chain, induces accumulation of ROS and causes DNA damage, while also impairing mucin 2 (MUC2)-mediated mucus barrier function, collectively driving the progression from colitis to CAC. **(B)** Vitamin B2 secreted by probiotics such as *Lactobacillus and Bifidobacterium exe*rts anti-CAC activity: vitamin B2 is converted into flavin mononucleotide (FMN)/flavin adenine dinucleotide (FAD), promoting ceramide synthase 3 (CerS3)-mediated sphingolipid synthesis; it also activates the epidermal growth factor receptor (EGFR)/PI3K/Akt/mTOR signaling axis, upregulates the expression of *c-Myc* and cyclin D1, thereby promoting proliferation and repair of intestinal epithelial cells, and ultimately inhibiting the initiation and progression of CAC. Created with Adobe Illustrator.

Hydrogen sulfide (H_2_S), which is a key gaseous signaling molecule derived from gut microbial metabolism, displays a notable dual nature in the pathogenesis of CAC (Lin et al., 2023). The primary sources of H₂S in the gut include sulfate-reducing bacteria (SRB) and the cysteine degradation pathway: SRB reduce sulfate (SO₄^2−^) to H₂S via dissimilatory sulfate respiration, while gut microbes (e.g., *Clostridium* and Enterobacteriaceae species) produce H₂S by metabolizing cysteine through key enzyme systems such as cysteine desulfurase and methionine γ-lyase (Munteanu et al., 2024). It is noteworthy that while SRB have been traditionally regarded as the primary H₂S producers, recent metagenomic studies indicate that the capacity for H₂S generation via the cysteine degradation pathway is widespread in the human gut microbiome, and its overall abundance is significantly higher than that of SRB (Braccia et al., 2021). The pathological effects of high-concentration H₂S manifest as multidimensional genotoxicity: by inhibiting mitochondrial cytochrome c oxidase activity, it disrupts energy metabolism and induces ROS burst, leading to oxidative stress; it directly acts on DNA molecules, causing strand breaks, base oxidation, and aberrant methylation modifications (Hanna et al., 2025) (Figure 4A).

Studies in animal models demonstrate that chronic high-level H₂S exposure impairs colonic epithelial barrier function by disrupting mucus layer integrity and diminishing Mucin 2 (MUC2) secretion (Blachier et al., 2019) (Figure 4A). A cycle emerges between barrier dysfunction and microbial dysbiosis, characterized by pathogenic overgrowth (e.g., *Fusobacterium nucleatum*), lipopolysaccharide release, and Toll-like receptor 4/NF-κB-mediated chronic inflammation, culminating in CRC initiation. In striking contrast, H₂S at physiological concentrations exhibits unique protective mechanisms: it upregulates the expression of tight junction proteins (zonula occludens-1, Claudin-1) to repair the physical barrier, and promotes goblet cell differentiation as well as mucin glycosylation (Bi et al., 2023). *In vitro* evidence demonstrates that H₂S donors, including diallyl disulfide, possess the capacity to reshape microbial biofilms, which enhances probiotic colonization resistance and curtails opportunistic pathogen expansion (Hu W. et al., 2022). This microbiota remodeling effect is closely linked to its metabolic regulation: H₂S modulates the bile acid metabolic profile, promoting an increased proportion of taurine-conjugated bile acids, which selectively enriches SCFAs-producing bacterial populations (Ma et al., 2019). In terms of immunomodulation microenvironment, H₂S remodels the immune tolerance network through epigenetic mechanisms: it activates ten-eleven translocation 1 and 2 dioxygenases to promote demethylation of the Foxp3 gene promoter region, thereby driving the directional differentiation of naïve T cells into Tregs (Yang et al., 2015). The expansion of functional Treg cells significantly inhibits the Th17 cell cell-mediated inflammatory response, reduces the activity of the IL-17/IL-23 axis, and thereby alleviates intestinal inflammation. At the clinical translational level, novel slow-release H₂S donors (e.g., GYY4137) have demonstrated dual efficacy in mucosal healing and anti-fibrosis in animal models of IBD (He et al., 2025). By sustainably releasing low concentrations of H₂S, they maintain anti-inflammatory effects while avoiding the toxicity associated with high concentrations. In summary, the role of H₂S in the intestinal microenvironment exhibits a clear concentration-dependent duality: at high concentrations, it promotes CAC progression through genotoxic and pro-inflammatory mechanisms; at low concentrations, it exerts protective effects by maintaining barrier function and inducing immune tolerance.

### 2.8 Vitamin B2 (riboflavin)

Vitamin B2 as an essential micronutrient for the human body, can be obtained not only from dietary intake but also through synthesis by the gut microbiota. Furthermore, it plays a multilayered protective role in the initiation and progression of CAC. Multiple commensal bacteria in the human gut are capable of synthesizing riboflavin, with genera such as *Lactobacillus* and *Bifidobacterium* being the primary producers (LeBlanc et al., 2013). They provide the host with a significant endogenous source of vitamin B2. Riboflavin, via its coenzyme forms flavin mononucleotide (FMN) and flavin adenine dinucleotide (FAD), inhibits ceramide synthase 3 (CerS3) activity to reduce very-long-chain ceramide synthesis, thereby promoting epithelial repair, while concurrently activating the EGFR-PI3K/Akt/mTOR signaling pathway to enhance intestinal epithelial proliferation and delay CRC progression (Qu et al., 2025) (Figure 4B). This regulatory effect has been validated in both the azoxymethane/dextran sulfate sodium (AOM/DSS) chemically induced mouse model and patient-derived organoids, suggesting that the riboflavin-CerS3 axis may represent a potential therapeutic target for CRC. Research has shown that riboflavin, as an essential precursor for the synthesis of key coenzymes (FAD/FMN), can significantly enhance the metabolic activity of butyrate-producing bacteria, thereby increasing intestinal butyrate levels (Liu L. et al., 2023). Analysis of clinical samples indicates that plasma riboflavin levels are positively correlated with the survival of CRC patients, suggesting its potential as a prognostic biomarker. Through the dual mechanisms of directly inhibiting cancer-associated enzyme activity and indirectly elevating the protective metabolite butyrate, riboflavin establishes an anti-inflammatory and anti-tumorigenic physiological state within the intestinal microenvironment.

### 2.9 Gut microbiota metabolites: a multidimensional regulatory hub for host immunity and epigenetics

Gut microbiota metabolites serve as core mediators linking dietary factors, microorganisms, and host physiology. On the one hand, SCFAs, secondary bile acids, and TMAO converge on the NF-κB/STAT3 signaling axis through complex crosstalk, collectively regulating the body’s inflammatory and proliferative states. SCFAs are primarily produced through the fermentation of dietary fiber. They exert anti-inflammatory effects by inhibiting HDAC and upregulating suppressor of cytokine signaling 3, thereby blocking the activation of NF-κB and STAT3 signaling (Gao et al., 2013; Peng et al., 2024). At physiological concentrations, secondary bile acids inhibit NF-κB via the TGR5 pathway; however, at high concentrations, DCA activates both NF-κB and STAT3 through oxidative stress (Jin et al., 2022; Lu et al., 2025). TMAO activates NF-κB through the PKC/ROS pathway and induces the expression of IL-6, which in turn activates STAT3. Under a high-fiber diet, SCFAs predominantly inhibit this axis to maintain homeostasis, whereas under a high-fat red meat diet, TMAO and excess DCA synergistically activate pro-inflammatory and pro-proliferative pathways (Seesaha et al., 2020).

On the other hand, these metabolites profoundly influence host gene expression and cell fate through multifaceted and multi-targeted epigenetic mechanisms, constituting what can be termed the “epigenetic axis.” The core of this axis lies in the ability of metabolites to enable precise regulation of pathophysiological processes, such as immunological homeostasis, barrier function, and tumorigenesis, by modulating key epigenetic mechanisms including DNA methylation, histone modifications, and chromatin remodeling. Butyrate, a classic HDAC inhibitor, stabilizes Treg differentiation by enhancing histone acetylation at the Foxp3 locus, while also inhibiting inhibitor of nuclear factor κB (IκBα) ubiquitination and degradation to block the NF-κB pathway (Furusawa et al., 2013; Kumar et al., 2009). Polyamine metabolites upregulate the expression of the tumor stem cell marker CD44 by inducing c-myc expression, which promotes histone acetylation in its promoter region, thereby enhancing tumor stem cell properties and inducing an immunosuppressive microenvironment. TMAO, by inhibiting DNA methyltransferase 1 activity, induces promoter demethylation of the tumor suppressor gene Secreted Frizzled-Related Protein 1, leading to aberrant activation of the Wnt/β-catenin pathway. Concurrently, it disrupts the function of α-ketoglutarate-dependent dioxygenases, perturbs the balance of histone methylation, and synergistically drives malignant tumor progression through AMP-activated protein kinase (AMPK)/mTOR-mediated metabolic reprogramming. H₂S promotes demethylation of the Foxp3 gene promoter by activating Ten-Eleven Translocation dioxygenases, thereby driving Treg differentiation and suppressing Th17 cell-mediated inflammatory responses (Yang et al., 2015).

In summary, the synergistic interplay between the convergence of signals across pathways and epigenetic regulation across multiple layers by gut microbiota metabolites collectively shapes intestinal immune homeostasis and the tumor microenvironment. This provides a theoretical foundation for dietary interventions in chronic inflammation and CRC.

## 3 Intervention strategies and therapeutic applications based on gut microbiota-derived metabolites

### 3.1 Intervention strategies

In response to the bidirectional regulatory roles of gut microbiota metabolites in CAC, current research is focused on the development of multi-level and multi-target intervention strategies. In terms of early warning, diagnostic models based on metabolite profiles are being developed. By detecting the levels of metabolites such as SCFAs, secondary bile acids, and TMAO in feces or serum, they aim to achieve early identification of CAC risk. Regarding interventional approaches, engineered bacterial targeted delivery systems are being employed to carry butyrate prodrugs, enabling their local release in the colon to enhance therapeutic efficacy and reduce systemic side effects (Sung et al., 2025). Meanwhile, bile acid sequestrants (e.g., cholestyramine) can bind bile acids in the gut, interrupting their enterohepatic circulation and thereby reducing the production and absorption of toxic secondary bile acids (Grundy et al., 1971). Dietary modification is equally crucial: increasing dietary fiber (e.g., fructans and resistant starch) promotes the proliferation of SCFA-producing bacteria (So et al., 2018); limiting red meat and high-choline foods helps reduce TMAO levels (Wang et al., 2019); supplementing with seaweed may, by providing sulfated polysaccharides, promote the colonization of beneficial bacteria such as *Bacteroides plebeius* and their subsequent butyrate production (Munoz-Munoz et al., 2021). These strategies collectively aim to reshape the balance of gut microbial metabolism, break the vicious cycle of “chronic inflammation → carcinogenesis” and provide new directions for the prevention and treatment of CAC.

### 3.2 Therapeutic applications

In the therapeutic field of CAC, probiotic intervention is a key and effective strategy. Probiotics are live microorganisms that confer a health benefit on the host when administered in adequate amounts. Multiple studies have confirmed that specific probiotics can modulate the structure of the gut microbiota, increase the synthesis of beneficial metabolites (e.g., SCFAs and indole derivatives), and inhibit the inflammatory-to-carcinogenic cascade. Common probiotics such as *Lactobacillus* and *Bifidobacterium* can increase short-chain fatty acid production, inhibit inflammatory signaling pathways like NF-κB, reduce the release of pro-inflammatory cytokines, and enhance the activity of anti-inflammatory factors, thereby comprehensively regulating the intestinal immune microenvironment (Ohashi and Fujisawa, 2023). For example, oral administration of *Bifidobacterium breve* lw01 can produce the metabolite indole-3-lactic acid, which activates the AhR in macrophages (Li Y. et al., 2024). This activation drives the differentiation of immature inflammatory macrophages into mature, homeostatic macrophages, thereby exerting tumor-suppressive effects. Oral supplementation with Limosilactobacillus fermentum GR-3 can reshape the gut microbiota structure, enhance intestinal epithelial barrier integrity, promote the production of beneficial metabolites (e.g., butyrate), and effectively alleviate intestinal inflammation, thereby inhibiting tumor development (Zhou et al., 2024). Although both *Bifidobacterium breve* and Limosilactobacillus fermentum can synergistically promote butyrate production and Treg differentiation, their mechanisms are distinct: The former is primarily responsible for driving cross-feeding by providing acetate and relies on direct interaction between cell wall peptidoglycan and Toll-like receptor 2 to induce IL-10-mediated immune tolerance; the latter is focused on providing lactate as a substrate and mainly regulates Tregs epigenetically through SCFAs by inhibiting HDAC or activating G protein-coupled receptors. This complementarity between “structural immune training” and “metabolic microenvironment remodeling” establishes the synergistic basis for their combined application (Bai et al., 2024). Based on the aforementioned complementary mechanisms, supplementation with a probiotic combination of *Limosilactobacillus fermentum* CECT5716 and *Bifidobacterium breve* CECT7263 in a randomized clinical trial involving preterm infants was found to be safe and effective in reducing the risk of severe necrotizing enterocolitis and improving feeding tolerance (Hurtado Suazo et al., 2025). Furthermore, they can activate MUC2 gene expression, increase mucus layer thickness, maintain intestinal barrier integrity, and inhibit the transition from inflammation to cancer (Engevik et al., 2019). Additional research indicates that *Saccharomyces boulardii* can precisely regulate the metabolic pathways of SCFAs, promoting their production (particularly butyrate) and suppressing the initiation and progression of CAC (Schneider, 2005). *Weissella cibaria* has been confirmed to effectively inhibit the initiation and progression of CAC (Hao et al., 2025). Its mechanism of action begins with the modulation of the gut microecology and remodeling of bile acid metabolism, which then specifically activates the intestinal farnesoid X receptor (FXR) signaling pathway, ultimately mediating a significant anticancer effect. In the chronic inflammatory microenvironment, CD8^+^ T cells exhibit a “double-edged sword” characteristic: while they can kill virus-infected or cancerous cells, certain subtypes also release large amounts of pro-inflammatory cytokines (e.g., interferon-γ and TNF-α) (Hua et al., 2025). These cytokines persistently damage tissues, exacerbate inflammation, and promote tumor development. The combined use of *Clostridium butyricum* and *Akkermansia muciniphila* can synergistically reduce CD8^+^ T cell numbers, thereby alleviating colitis and suppressing associated tumorigenesis. *Bacteroides plebeius*, which colonizes the human gut, is capable of degrading sulfated polysaccharides (e.g., sulfated glucan and carrageenan) from seaweeds and other marine products, and in this process produces the important metabolite butyrate (Chen et al., 2025). By virtue of this function, the bacterium essentially establishes a “butyrate factory” within the gut. By elevating butyrate levels, it effectively suppresses intestinal inflammation and enhances epithelial barrier function, thereby constructing a natural defense line against the development of colon cancer. The study suggests that supplementing with this probiotic and consuming dietary seaweed rich in sulfated polysaccharides may offer potential benefits for the prevention and treatment of inflammation-driven CRC. Unlike a single strain, the *Lactobacillus* cocktail preparation is a meticulously formulated mixture containing multiple *Lactobacillus* strains designed to enhance efficacy (Wang, 2025). It inhibits the initiation and progression of CAC by altering the intestinal metabolic environment, particularly by enhancing tryptophan metabolism and short-chain fatty acid synthesis. By combining together, they can simultaneously target multiple pathways, leading to a more comprehensive improvement in gut health. Following the discussion on the functional characteristics and intervention strategies of probiotics, prebiotics, which serve as their selectively fermented substrates, have become an indispensable component in this field of research due to their pivotal role in regulating gut microbiota homeostasis and synergistically enhancing probiotic effects. Prebiotics are a category of dietary components that are not digested by host enzymes but can selectively promote the growth and activity of beneficial gut bacteria. Supplementation with prebiotics such as inulin and fructooligosaccharides can significantly increase the abundance of short-chain fatty acid-producing bacteria, elevate SCFAs levels, and thereby improve gut barrier function while inhibiting inflammation and tumorigenesis (Hays et al., 2024). Beyond merely “feeding” the gut bacteria, a more advanced strategy involves directly “transplanting” a complete healthy microbial community. This approach, known as fecal microbiota transplantation (FMT), has been shown in numerous studies to have potential applications for treating IBD and preventing CAC (Song et al., 2024). FMT works by transplanting a “healthy” microbial ecosystem into a diseased individual, effectively reshaping the disordered intestinal microenvironment. It increases beneficial bacteria and reduces pathogens, thereby alleviating intestinal inflammation, repairing barrier function, and inhibiting abnormal cell proliferation. Ultimately, this blocks the malignant progression from “inflammation to dysplasia to carcinogenesis”. Although FMT holds promising potential in both therapeutic and preventive applications, its adoption as a long-term treatment or prevention strategy remains constrained by insufficient evidence, particularly concerning long-term safety and microbiota stability post-transplantation. A recent five-year follow-up study focused on an IBD-CAC cohort has contributed important data to this field, revealing that short-term adverse events associated with FMT are predominantly mild to moderate and self-limiting, with no observed increases in new malignancies or serious long-term risks during extended follow-up, thereby preliminarily confirming its long-term safety (Tian et al., 2022). However, the study also highlighted significant inter-individual variability in the persistence of donor microbiota engraftment, with only approximately half of the patients maintaining high microbial diversity by the end of the follow-up period (Staley et al., 2019). This finding suggests that achieving stable microbial reconstitution may necessitate strategies such as multiple transplantations or adjunctive interventions to consolidate therapeutic efficacy. Furthermore, the current clinical application of FMT continues to lack standardized systems for donor screening, preparation manufacturing, and quality control, which further limits its broader implementation in long-term management strategies.

In parallel with microbial interventions, drug development directly targeting metabolic pathways is also advancing. For instance, by binding to intestinal bile acids and interrupting enterohepatic recirculation, bile acid sequestrants suppress the production and limit the reabsorption of secondary bile acids (Feng et al., 2021). Engineered bacteria are utilized to deliver butyrate prodrugs for localized release in the gut (Xue et al., 2024). The butyrate then acts by upregulating tight junction proteins and activating the AMPK pathway, thereby repairing the intestinal barrier and exerting a therapeutic effect against CAC. UDCA, a hydrophilic bile acid, modulates bile acid signaling, reduces oxidative stress, and counteracts the damage caused by hydrophobic bile acids. Its therapeutic potential for CAC is currently being investigated. In conclusion, gut microbiota metabolites play a multifaceted role in CAC by functioning as anti-inflammatory agents that reduce intestinal inflammation, as anticancer agents that suppress tumor cell growth and dissemination, and as immunomodulators that reshape the immune microenvironment. These metabolites primarily contribute to the effective prevention and treatment of CAC through three key mechanisms: strengthening intestinal barrier function to fortify the gut’s ecological defense line; regulating cellular signaling pathways to precisely modulate cellular activities; and optimizing host–microbe interactions to comprehensively safeguard intestinal health. Current metabolite-based intervention strategies, including probiotics/prebiotics, FMT, and metabolite-targeting drugs, are progressively advancing from fundamental research to clinical exploration. Although challenges such as individual heterogeneity, causality confirmation, and clinical translation remain, these avenues undoubtedly open new paths for the precise prevention and treatment of CAC.

## 4 Discussion

Although significant progress has been made in understanding the roles of gut microbiota-derived metabolites in the pathogenesis and intervention strategies of CAC, this field still faces several key challenges and, simultaneously, presents multifaceted future opportunities. Current major challenges include individual heterogeneity and standardization hurdles. The composition of the gut microbiota and its metabolic profiles are influenced by a multitude of factors such as genetics, diet, environment, and medications, leading to significant inter-individual variation. This variability makes it difficult to establish unified standards for metabolite detection and intervention protocols, while also introducing uncertainty into efficacy prediction in clinical applications. A further challenge is the frequent dual, concentration-dependent nature of these metabolites. Compounds like H_2_S and secondary bile acids can play physiological roles at low concentrations yet drive pathology at high levels. This duality complicates therapeutic design, centering the challenge on achieving precise spatial and temporal concentration control within the diseased microenvironment. Furthermore, obstacles exist in clinical translation. These include issues of colonization resistance and stability for probiotic/prebiotic preparations in the human body; insufficient long-term safety data and lack of standardized donor screening for FMT; and uncertainties regarding the *in vivo* delivery efficiency, biosafety, and regulatory approval pathways for metabolite-targeting drugs, such as engineered bacterial delivery systems. In addition, the challenge of translating findings from animal models to humans is particularly prominent. There are significant species differences in the gut microbial composition between mouse models and human patients with CAC. For instance, bacterial families enriched in the mouse gut, such as Muribaculaceae, are present at very low abundance in the human gut, while families characteristic of humans, such as Prevotellaceae, are either absent or show vastly different abundances in mouse models. This discrepancy directly leads to inconsistencies in the types and concentrations of the gut microbial metabolic profile (e.g., SCFAs, secondary bile acids, H_2_S) between models and patients. Consequently, it is difficult to directly extrapolate the mechanisms of microbiota-metabolite-tumor regulation discovered in mouse models to humans, and it also limits the effective translation of intervention strategies targeting the gut metabolome from the preclinical stage to the clinical stage. Lastly, methodological limitations present a significant hurdle. While current technologies lack the sensitivity and coverage needed for complete gut metabolome profiling, a separate and distinct challenge is posed by the difficulty of achieving *in vivo*, real-time, and *in situ* monitoring of metabolite dynamics in the gut.

Looking ahead, research will advance toward precision and personalized medicine. By integrating multi-omics data with artificial intelligence models, it aims to construct individualized CAC risk prediction profiles, thereby enabling tailored intervention strategies based on each patient’s unique microbial features and metabolic phenotype. In terms of mechanistic dissection and technological application, cutting-edge techniques such as organoid-microbiota co-culture systems, single-cell sequencing, and spatial metabolomics can be employed to precisely dissect, at the molecular and cellular levels, how metabolites drive or suppress carcinogenesis through signaling pathways and epigenetic modifications. Meanwhile, advances in synthetic biology will propel the development of “living therapeutics,” such as engineered bacteria capable of sensing inflammatory signals and locally releasing therapeutic metabolites (e.g., butyrate). Regarding intervention strategies, the field will expand from traditional probiotics/prebiotics to next-generation probiotics (e.g., anaerobes) and novel-structure prebiotics, with their efficacy validated through rigorous clinical trials. It will also advance the standardization of FMT procedures and develop safer, more controllable synthetic microbiota transplantation products. Concurrently, efforts will be focused on creating more stable and precisely targeted metabolite prodrugs or receptor agonists/antagonists (e.g., farnesoid X receptor agonists). Interdisciplinary collaboration and clinical integration are crucial guarantees for achieving breakthroughs. This necessitates deep integration across fields such as microbiology, immunology, oncology, bioinformatics, clinical medicine, and bioengineering. Furthermore, incorporating the monitoring of microbiota-derived metabolites into clinical trial design is essential to accelerate the translation of basic research findings into clinical practice.

In conclusion, research on gut microbiota metabolites has opened new avenues for understanding and intervening in CAC. Despite formidable challenges such as individual heterogeneity and clinical translation, precision prevention and treatment strategies targeting gut microbiota metabolites hold the promise of ushering in new hope for patients with CAC in the future, through sustained technological innovation, in-depth mechanistic exploration, and interdisciplinary collaborative efforts.

## Glossary

CAC: Colitis-associated colorectal cancer
IBD: Inflammatory bowel disease
SCFAs: Short-chain fatty acids
TMAO: Trimethylamine N-oxide
STAT3: Signal transducer and activator of transcription 3
NF-κB: Nuclear factor κB
CRC: Colorectal cancer
TNF-α: Tumor necrosis factor-α
IL-1β: Interleukin-1β
IL-6: Interleukin-6
IL-10: Interleukin-10
ROS: Reactive oxygen species
IKK: IκB kinase
HDAC: Histone deacetylase
Th2: T helper 2
Tregs: Regulatory T cells
GPR43: G-protein coupled receptor 43
GPR109A: G-protein coupled receptor 109A
PKC: Protein kinase C
Nrf2: Nuclear factor erythroid 2-related factor 2
NLRP3: NOD-like receptor family pyrin domain containing 3
SUCNR1: Succinate receptor 1
HIF-1α: Hypoxia-inducible factor-1α
DCA: Deoxycholic acid
LCA: Lithocholic acid
UDCA: Ursodeoxycholic acid
TGR5: Takeda G protein-coupled receptor 5
FSP1: Ferroptosis suppressor protein 1
STAT6: Signal transducer and activator of transcription 6
PPAR-γ: Peroxisome proliferator-activated receptor γ
YAP: Yes-associated protein
AMPK: AMP-activated protein kinase
PI3K: Phosphoinositide 3-kinase
mTOR: Mammalian target of rapamycin
FMN: Flavin mononucleotide
FAD: Flavin adenine dinucleotide
CerS3: Ceramide synthase 3
AhR: Aryl hydrocarbon receptor
SRB: Sulfate-reducing bacteria
FMT: Fecal microbiota transplantation
AOM/DSS: Azoxymethane/dextran sulfate sodium
EGFR: Epidermal growth factor receptor
Akt: Ak strain transforming
CD44: Cluster of differentiation 44
MUC2: Mucin 2
